# Valedictory Address

**Published:** 1860-03

**Authors:** J. F. Jonston


### VALEDICTORY ADDRESS,

### TO THE GRADUATES OF THE OHIO COLLEGE OF DENTAL SURGERY.

BY J. F. JONSTON, D. D. S.

GENTLEMEN :—In accepting the invitation of the Faculty of this College to deliver to you as is the custom, an address, you can easily imagine that it would be with pleasureable emotions, when I tell you that just seven years ago I was placed as you are now. You can easily realize that my being here to-night would naturally awaken and bring vividly and fresh to my memory many pleasing incidents connected with the days of my pupilage in this institution—added to which the feelings of gratitude and pride, at the unmistakable evidence on the part of my professional parent, that her son has not been forgotten, but on the contrary is still remembered as entitled to her affections and esteem, would certainly be sufficient cause for feelings of exultation. I, on my part, am free to acknowlege that whatever of success I have met with professionally, is traceable most clearly to my own mind, to the thorough course of instruction and the good advice at parting she gave me. I am proud to acknowledge myself one of her sons, and the pleasure of congratulating and welcoming other young members of her family is enhanced on returning home, as I feel myself to-night, and find that age has but added strength and beauty to her. She is prosperous and has decked herself in an entirely new dress; but although that in which she nursed me has been cast off, there are enough remnants left to remind me of what she was, in the familiar faces of some of her pioneer professors, and how pleasant it is to find them. May they long be found at her side! But to drop the figure, gentlemen, we can not look with other feelings than those of pride upon this edifice, which is indeed an improvement upon that which occupied this ground when I was here a pupil; though that was a very comfortable old tenement, which at the time we thought answered very well, and which now we can not think of but with reverence. But this building indicative as it is of the healthy advancement of dental science and enterprise in the west, is especially worthy of your regard as being the only college owned by the profession, if I am correctly informed, in the world. It should be your aim now that you have a life interest in it, a sort of relationship in fact, to send your pupils to its support. The Ohio College of Dental Surgery must continue to flourish many, many years!

Having, gentlemen, been cut loose from the "leading strings" for the past seven years, in which time I have been floundering my way down life's stream, with nothing but my profession to steer by, I feel that I may with some propriety venture to offer to you who are just embarking, such advice as may be of advantage to guide you in a correct professional course—that at least may enable you the more easily to avoid the sand bars and difficult passages, that without a chart or a good pilot, so many are unable to pass, and either abandon the tortuous yet beautiful stream that leads to wealth and fame altogether, or eke out a miserable existence, stranded, when only half way. To be ambitious to excel in your calling, the desire to be popular as an operator, and to have the entire confidence of the community in which you reside, are feelings natural to every gentleman, and are when properly exhibited commendable traits of character. That we all possess them more or less, I fully believe. The poet has beautifully said with reference to this trait, (but I fear I can not do him justice as I quote from memory,) that—

> "The proud to gain it toils on toils endure,
> The modest shun it, but to make it sure;
> On globes and sceptres, nor on thrones it swells
> And trims the mid-night lamp in college cells;
> Nor ends with death, but nods in sable plumes,
> Adorns the hearse, and flutters on the tombs."

It is in the way these peculiarities are manifested, that good or bad results follow. That they are the natural incentives to all efforts when man is otherwise unembarrassed, no one doubts. You have given us an example of it in your praiseworthy endeavors to secure the triumph which is yours to-night. But aside from this natural love, this yearning for a respectable position, the pecuniary advantages arising therefrom will add material to this often latent, but never dying fire, and make it blaze forth in some form in all—that it may shed its genial warmth and light, in its most approved form in you, is the desire not only of the Faculty of this College, but of every true man, and none more than he who addresses you. To-night, perhaps, witnesses the last general meeting of the Graduating Class and Professors of this College for the present session, and to-night also severs the relationship of teacher and pupil. The feelings of both parties, no doubt, partake both of pleasure and pain. Pleasure that the work is accomplished—pain that friends must part, and pleasant associations be broken up. From the intimate acquaintance and relationship as your advisers and instructors, it is but natural that the Faculty should feel a deep interest in you, and having vouched for you to the public, they can not be other than anxious as to your course as professional men. They can do no more, however, than trust to your high sense of honor, which will make you especially careful not to be guilty of any professional delinquency that will in any wise compromise the character, or bring reproach upon, the institution whose highest honors you have received.

You must be conscious, gentlemen, that this night will be remembered, whilst your memory serves you, as the event of your life. From to-night you will date the beginning of your professional career, and scarcely a day will pass whilst in practice, but something will occur that will carry your thoughts rapidly back to your college days. Now the anxiety of passing creditably this ordeal is over, the channel of your thoughts is changed, and will naturally run into the future. The office arrangements, the location, and the numerous necessaries to equip you for the "battle of life," which is now about to commence with you in earnest, and the best and safest way to fight it, will be the all absorbing thought. And you will fully realize, now that you are separated from your instructors, and have received the significant appendage to your name of D. D. S., being thereby placed on terms of professional equality (as far as a title goes) with all in the profession—that you have suddenly emerged from the enquiring and attentive pupil, into the thoughtful, self-reliant, active practitioner. The change will appear greater to you than you can at this moment conceive; but when your first patient presents him or herself the full force of it will then burst upon you. The responsibility you have assumed and the impression you will have made toward your future success, can not be other than food for serious thought, until you are well established, and your reputation is a fixed fact. And to this end you must press determinedly on; let not the "grass grow under your feet" but "catch each transient hour," and "improve each moment as it flies," bearing in mind that

> "Life is a short summer, man a flower,
> He dies, alas, how soon he dies."

The profession you have chosen, is certainly not the most easy one, taken in any sense. Indeed to my mind, it is in several respects more difficult and arduous than others. Whilst in law or divinity if a man is a fair scholar, a clear reasoner and something of an orator, and he is industrious and studious, he is looked upon, and will pass for, more than ordinary. The same abilities in the practice of medicine, without the oratory, will enable the possessor to pass well. But in the practice of dentisty, you will find that it requires, not only constant toil, but you will have many weary and anxious days and nights—there are no two sides to our cases, no excuses for defeats. We bring in, it is true, always a bill of costs sometimes pretty heavy ones, but they are generally cheerfully paid, because our cases are, indeed *have to be*, more or less successes. At least they must always come up to what you promise your patient, but allow me to say just here, that it is by far the best plan not to raise their expectations too high. It is always more gratifying to have your operations prove more satisfactory than anticipated. To continue then, dentistry demands most of the requirements for other professions and in addition a large stock of firmness, gentleness, skillfulness, mechanism, science, art, and patience; of the latter we may safely say, *more* than any other. You, no doubt, have heard it remarked that, if Job had been a dentist, we should not have had so favorable an account of him. I am inclined to believe it. Every thing connected with the practice requiring the greatest nicety, the most perfect accuracy, the mind must of necessity constantly dwell upon that in which you are engaged. The work of many hours is totally ruined by one thoughtless or unlucky tap, and the result is disappointment to your patient and loss to yourself. A careless thrust may entirely unnerve your patient, ruin a frail tooth, and your reputation also, with the individual and his or her friends. In fact I know of no calling or profession, that requires the constant use of *all* the faculties so continuously as does dentistry. To keep up with the age you must patronize the literature of your profession, read and experiment, for "something new starts every day." You must watch and be active; for although you are graduates, I trust you will not soon entertain the idea, that you know enough. In a profession that is advancing as does ours, the oldest in it can find something to learn. If, therefore, your ambition leads you to attempt to reach its very summit, you will find it can only be attained through legitimate, industrious and persistent means. Give it this and many of the annoyances disappear, and the ascent appears less difficult. But the lazy, unscrupulous man will be in constant trouble, he will resort to all kinds of plans and experiments, to find an *easy* way, and will at last be found when old age shall have overtaken him, stumbling among the the many obstacles at the very foot of the hill, having with his increased burden of years even less courage to proceed than ever. Death finally calls and compels him to relinquish his but half finished task for ever. But it is nevertheless encouraging to think, that notwithstanding the difficulties named, the science of dentistry is so far perfected that the operations of a thoroughly competent man, can readily be distinguished from those of the pretender, and therefore it is that such an one will succeed, and be appreciated.

Dentistry, like the other professions, is becoming quite full, and the public are gradually learning how to choose the most reliable men from among its numerous applicants for their patronage. Every day we see the evidence, that more than ever practitioners must stand upon their merits, or fall. The public no longer takes a man for a dentist because he so advertises himself, they want at least to have some such evidence as you now hold, to even induce them to think of you as such. And I give you fair warning, gentlemen, that unless you are extremely lucky, and your lots be cast in very favorable places, where aching teeth abound, and where jaws are wide open, empty and begging to be filled with the "pearly gems," where there is no competition, and where in consequence your services will be in great demand, (and of such a locality I can not advise you, for I acknowledge myself ignorant of such a spot,) you must make up your mind to do a very small business at first. Practice will come in gradually. People are in these times very cautious; they are slow to entrust those valuable and truly sensitive organs, to the tender mercies of one, whose skill has not been tried. Indeed, you will be led to think your bell will never ring for a summons for professional services, whilst you will entertain no fears of the landlord ringing *punctually* at the end of the month. And even when a call shall have been made, for a tooth to be filled or even extracted, you will be conjecturing as to what kind of an impression you have made, whether the person will speak well or ill of you, and so on, with alternate hopes and fears, clouds and sunshine, will your practice be made and established. These difficulties which are attendant upon all professions when practiced upon correct principles, have frequently led young men to enter ours, (supposing it most available) and resort to empirical means to get at once into business. Having entered upon it for the sole purpose of making money rapidly and easily, and without any regard to those principles of honor, that any and every *man* should hold beyond prize, they rush head-long into excuses, stratagems and devices, to gammon a too credulous public. Succeeding, perhaps, for a short time, they continue their bombast until the merest booby will understand them. Their gasconade being unsubstantiated by good works, their utter unreliableness becomes patent—and from that time their reputation is made; and go where they may, some one will "turn up" who has seen them before, and will blast their prospects of humbugging by uttering for them too soon the word quack. But you, gentlemen, can be of good cheer. Your coming here is evidence to the public of your desire to be thoroughly qualified to discharge the duties of a practitioner, honestly, faithfully and skillfully. That you did not skulk your duties as students, your diploma corroborates. You now have but to temper your zeal with prudence to succeed; and yet I could not conscientiously leave this part of my subject without urging upon you the absolute necessity of continued study and close observation. By so doing you will certainly improve, and in every succeeding operation you will discover that it has been performed with more ease and is yet far better than the preceding one. The artist—

let him be sculptor, painter, or what not, is ever trying to improve on former efforts, knowing well that he can arrive at eminence only by so doing, and that his works being likely to meet the eye of other artists, their criticisms will make either for or against him. How much greater is the necessity for untiring effort on the part of the dentist. His operations are equally liable to be judged by his professional brethren, and their verdict will tend either to build him up or cast him down, and what is of greater consequence, if the operation is not properly performed, it is a sting to his conscience, for his patient must suffer either pain or loss through his defects. Either contingency could not be other than deploring. For these reasons I have many nights lain awake studying how best to succeed in difficult cases. Yes, and must acknowledge to having prayed to Him, who is the giver of all good, to strengthen my understanding, and endow me with the necessary faculties to discharge my duties as an efficient practitioner.

I lay no claims, gentlemen, to unusual honesty, or *any* piety, (and yet I can not but believe that "Prayer ardent opens heaven")—it was most likely because I had been taught that "honesty is the best policy." But when a young man is thrown entirely upon his own resources, having nothing save his profession to rely upon, nothing being left after College expenses are paid, for a "rainy day; feeling his entire wealth consisted in his profession and a good character, to sustain which in this unfriendly world, money enough to pay promptly all debts contracted is indispensable, to obtain which he must have business; and when he has determined (as I trust you have) to succeed, if at all, by honorable means only, and when he shall have married (as all men of taste will) a beautiful, tender and loving girl, and desires, as all who are worthy of the name of men should, to keep her comfortable and happy; when he shall have learned that the public will sift him thoroughly and discover the amount of chaff in him; and when, further, he will have learned that there are among his competitors men who are in every respect as professional men his equals, (for thanks to such institutions as this, good dentists are not so scarce as they were previous to their existence); and with a perfect knowledge of the fact that defective dental operations, if he were content to make such, can not long remain undiscovered,—it would be strange if a man should not pray to be made equal to the task. Gentlemen, I do not allude to these things to discourage you. I speak of them merely to show that difficulties beset this, as other professions, and that the pretender can not long successfully combat with the competent man. With you, who have qualified yourselves, they should be regarded as words of encouragement. "Put your best foot foremost," and patiently and faithfully adhere to the correct principles of practice as taught you here, and you will not only conquer all obstacles, but earn a reputation that your children (after you have long since "gone to your rest,") will speak of with pride. As before intimated, I entertain no fears for you. You have honorably won your position to-night, which is proof enough that you are not of that make that will yield in future contests.

Now, having pointed out some of the difficulties to be met, it would, perhaps, be nothing more than proper I should call your attention to certain necessary precautions in a more specific manner than I have done. I shall not, however, dwell for a moment upon methods of practice—of that you have heard in these halls from more competent sources, but shall invite your attention to those matters appertaining more especially to your deportment as professional men, and those little attentions to your person and apartments, that, added to a thorough knowledge of performing operations, give popularity and character to yourself and establishment. First, then, as to advice, that being an article very freely bestowed upon dental patients. Did you ever hear of any person being influential in teaching doctrines they themselves totally disregarded? Must it not, then, be acknowledged that advice from a dentist to his patient with reference to absolute cleanliness being indispensable to preserve the teeth, comes with exceedingly bad grace from one who makes of his buccal cavity a receptacle for pig-tail tobacco and other vile preparations of " the weed," and of his teeth, a patent self-adjustable portable mill to grind it, and who seldom or never uses the brush he so learnedly recommends? Do you think such advice would be heeded by children, who would be most benefited? It is true that his bringing himself in such close contact might so disgust sensitive people, that they would be especially careful to avoid being in such a condition themselves; but it would certainly be vastly more agreeable to teach them by an example of the opposite condition of things, to at least have that decent regard for the feelings of others that should prompt every operator, who has the least self-respect, to keep his teeth and breath, as well as his finger nails and hands, unexceptionably pure.

I knew a dentist, who, I am happy to say, is not a graduate of this or any other dental College, who chewed " the weed " incessantly, and if perchance he had but just taken a " chaw " when a patient might happen to enter, he was much too careful to throw it away; but would stow it with great skill, into either the cavity of an old molar, or that of his cheek. Having an unexpected call to fill a tooth for a young lady, after having but just bitten off a piece of Cavendish, he hastily made the usual deposition of it, and proceeded to business. The tooth being rather a difficult one to fill, he in a great effort relaxed his hold upon the tobacco, which gracefully rolled out into her wide-open mouth. The spitting and spattering on her part, and the apologizing on his, can be imagined, but not described. This same individual was innocent of ever having provided himself with (to him) those superfluous articles termed napkins. If he found a plug was going to be submerged, and he thought it might be injured thereby, his yellow " nose wiper " was at once brought to the rescue. In the recital of this case, I have not drawn upon my imagination. The facts were given me respectively by a relative of the individual, and a young lady who was an eye-witness. The result of such unwarrantable neglect of the rules of common decency, caused him to suppose he was not properly appreciated, and he has accordingly long since retired from the profession to the more congenial pursuits of a farmer, where his peculiar tastes can be indulged with less unpleasant consequences.

I believe I never had but one patient that would have felt *comfortable* under such treatment as here spoken of; and he presented himself about two months since. He was from a neighboring village, and had been attending the University. He desired his teeth thorougly examined, and such as needed filling, filled. Wishing to have a very critical examination made, he remarked that his teeth were not clean enough; he had been in the habit of using his finger, but thought it did not answer for a case like the present; therefore, would I lend him a tooth-brush? I acknowledge this tried my nerves, for although I had heard of the demand for the public tooth-brush, on the steamboat, I never expected to see a live specimen of the same sort in my office. I handed him a glass of water, and apologized for not being provided for such emergencies, as I best could. On speaking of this occurrence to a friend, he remarked that he knew a dentist, who always gave his patients' teeth a vigorous brushing, with a brush he kept for that purpose, well covered with his double back-action tooth powder, as a finishing process after removing tartar. I would advise you not to adopt this mode of practice. On the contrary, your forceps and all other instruments, in short, every thing that comes in contact with the teeth, should be well washed after using, and every particle of blood or other substance that could taint or soil them, be carefully removed. Attention to this matter costs very little trouble, preserves your instruments in good order, and adds very materially to the popularity of your establishment. For as has been shown in the rather aggravated cases spoken of, it is quite too much neglected. An apparatus for washing the hands, well supplied with water, soap and towels, should be sufficiently near, that the patient waiting for your services may be assured that the very necessary act of ablution has been attended to; for judging others' feelings by my own, I should think they would become quite nervous if they thought it had not. And yet I have known dentists who will operate all day without thinking of it; but they never had that kind of practice which I should consider desirable.

To be able to receive your patients in such a manner as to make them feel as soon as possible on friendly terms, and thus prepare them for those more intimate relations, when they take the chair, and further, to be able to drive off in a measure the pain and other annoyances of an operation, by diverting the mind with conversation, if you find it agreeable, as it most frequently will be, if properly chosen, and there is not *too much* of it,—are accomplishments you should cultivate. The advantage, then, of employing your leisure hours, not only in reading up your dental literature, but the current events of the day, and such standard works as your time will permit of, is apparent. And it is not only necessary to make you agreeable in your office, but out of it also. The dentist of our days has so far advanced in the public estimation as a professional man (and we may again thank our Colleges for it), that he is regarded as a worthy companion, and associate for any class of society. And when we reflect upon the influence of wealth and titles, even in this free republic, it is no mean compliment.

I advise you to cultivate friendly relations with your competitors, that is, if they are not quacks. If they are, have the courage to say so, and avoid them; but never let the "green-eyed monster" occasion you to speak ill of or treat disrespectfully a worthy man. Unfriendly relations with such are always supposed to arise from jealousy, and they no doubt most frequently do. But this, besides doing no good (rather an injury) is an evidence of weakness; indeed, shows clearly a want of confidence in yourself, otherwise you could have but little in a professional way, to be jealous of. Neither will it advantage you to speak ill of another's operations. The person having come to you, the former dentist still being comeatable, is enough to show that dissatisfaction exists. You have but to see that what you may do is well done, otherwise, the compliment might be returned.

Your relationship to the medical profession should be a matter worthy of thought and attention. It has been too much the custom, at least in so far as my observation has been extended, for the dentist to yield entirely too much to the dictation of medical men. It is quite frequently the case that the services of the one are required conjointly with those of the other ; and while I would have you refrain from putting on professional airs, or using with too much precision professional terms, or making in the least any pedantic display, I would have you feel sufficiently conscious of your ability, to decide for yourself what the nature of your services shall be, in so far as the mode of operating is concerned, at all events. Sometimes a medical man will show considerable jealousy towards the dentist, and to convince the patient, that needs the services of both, that no one knows any thing but himself, will dictate with as much impudence as ignorance, what the dentist shall do. If he is right in his directions, of course follow them, if he is wrong, operate only in conformity with your judgment, or give up the case. For if you obeyed his instructions, and any unpleasant consequences followed, such a one would not fail to try to saddle you with the blame and responsibility. Be then especially careful not to *infringe* upon their rights, and always maintain your own.

It is due, however, to candor and justice to say, that while you will undoubtedly find in the mother profession many such ignoramuses as just alluded to, yet the great majority are qnite the reverse. They are gentlemen, who will respect your rights, and in proportion to their own attainments, will the more cheerfully acknowledge yours. Indeed, the best physicians almost invariably turn over to the dentists all cases that properly come under their supervision. Neither are they slow in acknowledging their superior knowledge of such cases, not only to them personally, but to their patients. As an example of this, the best surgeon, perhaps, in the city I live in, was called about midnight, four months since, to attend a woman who had been having teeth extracted at my office the afternoon previous, and who was bleeding severely. The Doctor applied some tannin on a roll of linen, tied up her jaws, and told her to keep still as possible until morning, and he thought all would be right. When morning came, however, she was yet bleeding, and together with her friends, had become much alarmed, and sent early for me. On learning that the case was under treatment by the Doctor, I of course refused to interfere, telling them he was able (which was a fact) to attend to it. Shortly after the Doctor called, and requested me to take the case in hand, remarking that I was more familiar with such difficulties, and could, therefore, manipulate to better advantage, and he had so informed the patient. I then felt at liberty to attend to it, which I did, and sent the lady home (she lived some forty miles away) the next day to her husband, who being a physician, on learning of the trouble, she since informed me, gave her a scolding for not knowing enough to send for me at first. This is not an uncommon case. I dare say the like has occurred to others. It only shows the views of practical medical men in regard to this matter. Indeed, it is now regarded by all intelligent people to be just in as bad taste for a physician to practice any branch of dentistry, as it would be for a dentist to treat fevers. The public condemn them alike, and I have yet to see the man who succeeded well, who has attempted to practice the two together.

Gentlemen, you have the right to assume the position of master of your profession, and should do so, both in justice to yourselves and your Alma Mater, who has given you your credentials to-night so to do. And just in so far as you follow the teachings of that Book, which you have received with your Diploma, and "do unto others as you would they should do unto you," you will not overstep the bounds of propriety. And further, I indulge the hope that the time will arrive when the use of the letters D. D. S. in a professional sense will be as well and generally understood as are those of M. D., and that the public will be as anxious to find out whether a young dentist is a D. D. S., as they now generally are to learn if a physician is an M. D. And although I think it is in *very bad taste* for gentlemen to *parade* their titles before a community, yet I believe it to be the duty of graduates of all dental schools to use this abbreviation in advertising a card, which they can very easily do, without using the word dental, and still in an unassuming way very clearly indicate their business. Medical men consider it no disgrace, no breach of etiquette, to use the M. D., when entitled to it. Why should it be for us to use our D. D. S., under the same circumstances? The only objection ever urged is, that it is not understood. The plan I propose would *make* it understood, and we should soon see it classed among the abbreviations in the Spelling Book. If it is never to be understood, if it is to give no distinction, there is no use in having it. I, for my part, am quite as proud of my title as is the physician of his. It costs quite as much, and gives us quite as much trouble to obtain it, and it is one letter longer. I reiterate the opinion, that if all the graduates, and those who for good reasons have had the honor conferred, would use it, but *invariably with discretion*, the public will soon understand what it means, and use it to as great an extent in discriminating between reliable and unreliable practitioners, as they undoubtedly do among physicians. When this can be attained, as I believe it may, all genuine men will rejoice, and none more than those old pioneers, who have never enjoyed the privileges that are now yours, and yet have labored unceasingly to this end. If you could but realize the difficulties they encountered, even the scoffing and jeers of many who supposed they were practicing almost a vagrant's calling, you could not be otherwise than grateful to them and such institutions as this, for raising your profession up to the standard of the oldest, in point of respectability and learning. And I think further, that no young man that enters the profession hereafter, should feel content, or think of practicing longer than he can possibly help, without having obtained, as you have done, a diploma. There is no longer any excuse for such.

With reference to your office, furniture, instruments, etc., etc , I can not advise you, other than to obtain under all circumstances those that are neat and most useful. Further than that is a matter of taste, in which you will be governed by your own opinions, the community you practice in, and more especially the condition of your port-monaie. Poor Richard says, " Ere fancy you consult, consult your purse." The advice is pertinent.

But I weary you, and must close. I rejoice with you, that your most ardent wish has been realized. You have passed your examination, and have graduated with honor, and now hold in your possession the attestation of the Faculty of the Ohio College of Dental Surgeons to that effect. And now I have the pleasure of congratulating you, upon your triumphal entry into the ranks of the legitimate practitioner of dentistry, and with no other emotions than those of pleasure, extend to you in their behalf, the right hand of fellowship. Be but as zealous in the faithful discharge of your duties as professional men, as you have been in your studies to obtain to-night's distinction, and the additional diploma of " Well done thou good and faithful servant," will be yours.

